# Prognostic Significance of Diastolic Dysfunction in Type 2 Diabetes Mellitus Patients With Sepsis and Septic Shock: Insights From a Longitudinal Tertiary Care Study

**DOI:** 10.7759/cureus.45894

**Published:** 2023-09-25

**Authors:** Nonita Thockchom, Mukesh Bairwa, Ravi Kant, Barun Kumar, Yogesh Bahurupi, Bela Goyal

**Affiliations:** 1 Internal Medicine, All India Institute of Medical Sciences, Rishikesh, IND; 2 General Medicine, All India Institute of Medical Sciences, Rishikesh, IND; 3 Cardiology, All India Institute of Medical Sciences, Rishikesh, IND; 4 Community and Family Medicine, All India Institute of Medical Sciences, Rishikesh, IND; 5 Biochemistry, All India Institute of Medical Sciences, Rishikesh, IND

**Keywords:** type 2 diabetes mellitus, icu mortality, 2d echocardiography, left ventricular diastolic dysfunction, septic shock, sepsis

## Abstract

Background: Sepsis is one of the leading contributors to global mortality and morbidity, causing multi-organ failure, mainly involving cardiovascular failure, both systolic and diastolic dysfunction, leading to adverse clinical outcomes. There is little clinical data on the correlation with the mortality of patients with type 2 diabetes mellitus (T2DM) with sepsis and septic shock and left ventricular diastolic dysfunction. Our study sought to assess whether the severity of diastolic dysfunction could predict 28-day mortality.

Methodology: The study included T2DM patients admitted to the intensive care unit (ICU) with sepsis and septic shock defined according to the Third International Consensus Definitions for Sepsis and Septic Shock at a tertiary care center in northern India. A total of 132 patients (age = 61.01 ± 13.12 years; 62% male; mean APACHE II (Acute Physiology and Chronic Health Evaluation II) score = 25.74 ± 4.79; Sequential Organ Failure Assessment (SOFA) score = 12.34 ± 3.36) underwent transthoracic echocardiography within two hours of ICU admission till 28 days of admission or till mortality occurred. Clinical variables (APACHE II and SOFA score) and cardiac biomarkers, such as N-terminal pro-B-type natriuretic peptide (NT-pro-BNP), troponin I, and creatine phosphokinase-MB, were measured at the time of admission and after 72 hours to compare with mortality. Diastolic dysfunction was defined according to the American Society of Echocardiography (ASE) 2009 guidelines, classifying subjects into grade 0 (normal), if early diastolic velocity (e') ≥ 8 cm/s; grade 1 (impaired relaxation), if e' < 8 cm/s and early (E) to late (A) ventricular filling velocities (E/A) ratio < 0.8; grade 2 (pseudo normal), if e' < 8 cm/s, E/A = 0.8-1.5, and peak E-wave velocity by the peak e' velocity (E/e') ratio = 9-12; and grade 3 (restrictive), if e' < 8 cm/s, E/A > 2, deceleration time (DT) < 160 ms, and E/e' ≥ 13.

Results: Thirty-seven (40.65%) out of 132 patients had diastolic dysfunction on initial echocardiography, while 54 (59.34%) had diastolic dysfunction on at least subsequent echocardiography. Total mortality was 68.93% with the highest mortality (100%) observed among those with grade 3 diastolic dysfunction. The 28-day mortality with diastolic dysfunction in sepsis and septic shock patients showed significant results (p < 0.001), indicating that with a higher E/A ratio or higher grade of diastolic dysfunction with the increase in SOFA score, the early ICU mortality is the highest and have the shortest duration of ICU stay with mean ± SD = 6.2 ± 2.48, as compared to other grades with 100% mortality. Also, the cardiac biomarker NT-pro-BNP was markedly elevated with a mean ± SD value of 503 ± 269.3 pg/ml, indicating early predicted mortality. No correlation was detected between mortality and the mean levels of fasting blood sugar, postprandial blood sugar, and glycosylated hemoglobin.

Conclusion: Our study concluded that diastolic dysfunction is an important and strongest independent mortality predictor in patients with T2DM with severe sepsis and septic shock, and the higher the grade of diastolic dysfunction, the higher the mortality with the lowest mean ICU stay.

## Introduction

Sepsis affects 48.9 million people per year globally, of which 11 million die, representing a mortality rate of 19.7% [1]. According to the Third International Consensus Definitions for Sepsis and Septic Shock (Sepsis-3), "sepsis is defined as a life-threatening dysfunction caused by the dysregulated host response to infection." It is associated with a >10% in-hospital mortality. "Septic shock is defined as the subset of sepsis in which particularly profound circulatory, cellular, and metabolic abnormalities are clinically identified by a vasopressor requirement to maintain a mean arterial pressure of 65 mmHg or greater and serum lactate level greater than 2 mmol/L (>18 mg/dl) in the absence of hypovolemia with a mortality rate of >40%" [2]. Cardiovascular failure due to sepsis involves peripheral vascular dysfunction and myocardial dysfunction [3]. There is decreased systolic contractility that leads to low ventricular ejection fraction and stroke volume. So, initially, this decrease in stroke volume is compensated by the increase in diastolic filling during volume resuscitation and decreased after-load due to septic arterial dilatation [4]. A combination of both systolic and diastolic dysfunction leads to adverse clinical outcomes [5,6]. In the last few decades, the incidence of diabetes mellitus has increased rapidly, especially in low and middle-income countries like India [7,8]. We, therefore, conducted a study among type 2 diabetes mellitus (T2DM) patients hospitalized in the intensive care unit (ICU) with sepsis and septic shock to find out the prevalence and grade of left ventricular diastolic dysfunction (LVDD) and its correlation with the mortality of the patient.

## Materials and methods

This observational cohort study was conducted at a tertiary care center in northern India over 18 months from July 2022 to November 2022 after obtaining approval from the Institutional Ethics Committee, All India Institute of Medical Sciences, Rishikesh (AIIMS/IEC/2/321). T2DM was defined based on the American Diabetes Association (ADA) criteria, which include an elevated glycated hemoglobin A1c (HbA1c) level of 6.5% or higher, a random blood sugar (RBS) measurement equal to or exceeding 200 mg/dL, a fasting blood sugar (FBS) reading of 126 mg/dL or higher, and an oral glucose tolerance test (OGTT) result that reveals a two-hour glucose level in venous plasma of 200 mg/dL or greater. All subsequent patients aged > 18 years having T2DM (ADA criteria) with sepsis and septic shock (according to surviving sepsis guidelines) were included. Severe sepsis was defined as having any of the following: (i) evidence of an infection or clinical suspicion of infections; (ii) leukocytosis or leukopenia with total leucocyte count <4000 or >12,000; (iii) tachypnea with respiratory rate > 22/minute or needing mechanical ventilation; (iv) systolic blood pressure < 90 mmHg; (v) temperature > 38°C or < 36°C; or (vi) any evidence of any end organ failure. Septic shock was defined as "severe sepsis with severe hypotension after failed fluid resuscitation lasting for more than one hour, or requirement of a vasopressor support" [2]. Patients with known cases of valvular heart disease, with two-dimensional echocardiography, cardiac evidence showing regional wall motion abnormality suggestive of ischemia or previous myocardial infarction, and with poor quality echocardiographic images and measurement were excluded from the study. The primary objective of the study was to study diastolic dysfunction and 28-day mortality in patients with sepsis and septic shock. Secondary objectives included the study of clinical variables (Sequential Organ Failure Assessment (SOFA) scores and APACHE II (Acute Physiology and Chronic Health Evaluation II) scores) and biochemical (FBS, postprandial blood sugar (PPBS), and HbA1c) and cardiac biomarkers (N-terminal pro-B-type natriuretic peptide (NT-pro-BNP), troponin I (Trop-I), and creatine phosphokinase-MB (CPK-MB)) with mortality in patients with sepsis and septic shock.

Clinical data

All demographics and clinical parameters, daily fluids resuscitation, vasopressor requirement, hematological and biochemical data, including FBS, PPBS, and HbA1c, arterial lactate levels, sepsis biomarkers, cardiac biomarkers (NT-pro-BNP, CPK-MB, and Trop-I), blood cultures, and cultures of specimens from the site of infection were measured since the time of diagnosis of severe sepsis or septic shock and on subsequent days of echocardiography and the mean, minimum, and maximal were calculated. Disease severity by SOFA and APACHE II scores was assessed on the day of ICU admission, 72 hours after admission, and soon to 28 days after admission or till mortality occurred.

Echocardiography

**Figure 1:**
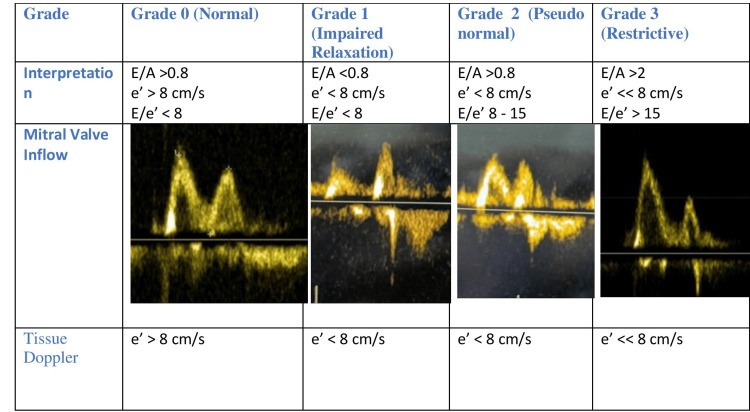
Classification of diastolic dysfunction. Diastolic dysfunction was defined according to the American Society of Echocardiography (ASE) 2009 guidelines [9].

All patients underwent serial transthoracic echocardiography using an echocardiographic machine, with an echo probe of 2-4 MHz. The initial echocardiogram was done after the diagnosis of sepsis in the ICU. The subsequent echocardiogram was performed on the next day to confirm the stability or any changes in the echocardiogram. Diastolic dysfunction was assessed by measuring E and A peak velocities using spectral Doppler of mitral inflow and e’ and a’ velocities using tissue Doppler of the septal mitral annulus in the apical four-chamber view. We defined diastolic dysfunction according to the American Society of Echocardiography (ASE) 2009 guidelines, classifying subjects into grade 0 (normal), if e' ≥ 8 cm/s; grade 1 (impaired relaxation), if e' < 8 cm/s and E/A < 0.8; grade 2 (pseudo normal), if e' < 8 cm/s, E/A = 0.8-1.5, and E/e' = 9-12; and grade 3 (restrictive), if e' < 8cm/s, E/A > 2, deceleration time (DT) < 160 ms, and E/e' ≥ 13 (Figure 1) [9]. Echocardiogram showing valvular heart disease, evidence of regional wall motion abnormality suggesting regional ischemia or previous myocardial infarction, and patients with poor-quality echocardiographic images and measurements were excluded from the study.

Statistical analysis

The data obtained were entered in MS Excel 2016 (Microsoft Corporation, Redmond, WA) and analysis was done using MS Excel 2016 and SPSS version 23 (IBM Corp., Armonk, NY). Assessment of diastolic dysfunction and its correlation with the 28-day mortality of the patient was done by chi-square test. The SOFA and APACHE II scores were analyzed by the Mann-Whitney test. The inotropes requirement was analyzed by Pearson's chi-square test. The cardiac biomarkers Trop-I, CPK-MB, and NT-pro-BNP were analyzed by the Mann-Whitney test. FBS, PPBS, and HbA1C were analyzed by unpaired Student's T-test. All the categorical variables are reported as percentages and continuous or discrete variables are reported as means ± standard deviation (SD). A p-value < 0.05 was considered significant.

## Results

A total of 132 patients were enrolled after the exclusion of 12 patients with poor echocardiography images, 10 patients with known cases of coronary artery disease showing regional wall abnormalities, and five patients with moderate to severe valvular heart disease. The main source of sepsis was community-acquired pneumonia (41.60%), complicated urinary tract infections (21.20%), hospital-acquired pneumonia (12.80%), gastrointestinal infections (6%), skin and soft tissue infections (4%), and others (14%). The source of positive infections was confirmed by culture-positive organisms isolated in 86 (65.15%) patients, of which, the most common organisms were *Acinetobacter baumannii* (26.5%), *Escherichia coli* (16.6%), *Klebsiella* (12.80%), *Pseudomonas* (6.80%), and *Streptococcus pneumonia* (2.20%). A total of 88 patients (66.6%) experienced septic shock despite fluid resuscitation requiring inotropes: single inotropes, i.e., norepinephrine (12, 9.09%), dual inotropes, i.e., norepinephrine and vasopressin (69, 52.27%), and triple inotropes, i.e., norepinephrine, vasopressin, and dobutamine/dopamine (61, 46.2%) (Table 1).

**Table 1:** Comparison of demographic clinical and biochemical data between survivors and non-survivors. NT-pro-BNP: N-terminal pro-B-type natriuretic peptide; SOFA: Sequential Organ Failure Assessment; APACHE II: Acute Physiology and Chronic Health Evaluation II; SD: standard deviation; CPK-MB: creatine phosphokinase-MB; BP: blood pressure; ICU: intensive care unit; Trop-I: troponin-I; Sao2: oxygen saturation; FBS: fasting blood sugar; PPBS: postprandial blood sugar; Hb: hemoglobin; HbA1c: glycated hemoglobin.

	Survivor (n = 46)	Non-survivor (n = 86)	P-value
Age (mean ± SD)	57.43 ± 11.29	61.01 ± 13.12	0.120
Gender, female %	16 (34.8%)	33 (38.4%)	0.684
Single vasoactive	9 (19.6%)	3 (3.5%)	0.004
Dual vasoactive	18 (39.1%)	51 (59.3%)	0.004
Triple vasoactive	19 (41.3%)	32 (37.2%)	0.004
NT-pro-BNP (pg/ml)	286.40 ± 206.9	503.24 ± 269.3	0.0001
Mean CPK-MB (IU/L)	5.89	5.12	0.031
Mean ICU stays (days)	12.65	9.07	0.055
Mean APACHE-II score (at the time of admission)	18.48	20.26	0.0001
Mean APACHE-II score (after 72 hours)	22.09	25.74	0.0001
Mean SOFA score (at admission)	8.70	8.91	0.517
Mean SOFA score (after 72 hours)	10.89	12.34	0.0001
Mean heart rate (b.p.m.)	84	88	0.521
Mean systolic BP (mmHg)	111.47	102.23	0.423
Mean diastolic BP (mmHg)	77.11	70.52	0.463
Lowest Hb (g%)	8.4	4.2	0.012
Lowest SaO2 (%)	89.24%	87.64%	0.120
Lowest pH	7.12	7.086	0.001
Creatinine (mg/dl), max.	3.07	5.07	0.120
Positive Trop-I	14 (30.4%)	32 (37.2%)	0.436
FBS (mg/dl), mean ± SD	164.2 ± 68.9	173.5 ± 70.1	0.466
PPBS (mg/dl), mean ±SD	279.2 ± 66.9	293.5 ± 69.1	0.254
HbA1c (%), mean ± SD	8.3 ± 1.2	8.5 ± 1.3	0.388

All enrolled patients were under mechanical ventilator at the time of echocardiography due to respiratory dysfunction or failure or low Glasgow Coma Scale score. In all the patients, after admission to the intensive care unit, echocardiography was performed; 37 (40.65%) had diastolic dysfunction at the time of initial echocardiography and 54 (59.34%) had diastolic dysfunction on subsequent echocardiography. The diastolic dysfunction parameters were assessed, left ventricular ejection fraction was calculated, and the studies were averaged and used for further analysis.

Predictors of mortality

A total of 132 patients were enrolled in this study, of which 88 (66.67%) died in the ICU and 44 (33.3%) survived. Among the non-survivors, the highest mortality was found in patients with grade 3 of LVDD. Patients who had normal LVDD showed the lowest mean ICU stay (6.26.2 ± 2.48) as compared to those who had grade 2 LVVD (9.07 ± 4.06) and grade 3 LVVD (12.65 ± 10.68), respectively.

The mortality at 28 days was analyzed by chi-square test, which shows 13 (100%) mortality in grade 3 LVDD, 19 (86.37%) mortality in grade 2, 41 (73.22%) in grade 1, and 15 (36.7%) in normal LVDD patients, suggesting that rise in mortality at 28 days as the grades of diastolic dysfunction increase indicates diastolic dysfunction is a major important indicator for early mortality in sepsis (Table 2).

**Table 2:** Echocardiographic comparison between survivors and non-survivors. E/A: peak mitral velocity at early diastole and late systole; DT: deceleration time; LVEF: left ventricle ejection fraction; LVDD: left ventricular diastolic dysfunction.

	Survivor (n = 46)	Non-survivor (n = 86)	P-value
Isovolumic relaxation time (ms)	48.56 ± 22.69	60.56 ± 53.56	0.016
E wave (cm/s)	94 ± 30	88 ± 21	0.175
A wave (cm/s)	85 ± 50	76 ± 22	0.012
E/A ratio	1.21 ± 0.45	1.20 ± 0.56	0.89
E wave deceleration time (ms)	145 ± 45	142 ± 45	0.67
Septal s′ (TDI, cm/s)	11.0 ± 2.8	8.6 ± 3.0	0.001
e′ (TDI, cm/s)	10.3 ± 3.3	6.7 ± 2.1	0.0001
a′ (TDI, cm/s)	9.8 ± 3.3	8.4 ± 4.1	0.010
Lateral s′ (TDI, cm/s)	11.4 ± 3.6	9.6 ± 3.7	0.001
e′ (TDI, cm/s)	11.2 ± 4.2	9.1 ± 3.4	0.001
a′ (TDI, cm/s)	10.2 ± 3.5	8.5 ± 4.3	0.012
LVEF (%)	55 ± 8	57 ± 12	0.14
Grade 1 LVDD	17 (37%)	39 (45.3%)	0.001
Grade 2 LVDD	3 (6.5%)	19 (22.1%)	0.001
Grade 3 LVDD	0 (0%)	13 (15.1%)	0.001

The mean SOFA and mean APACHE II scores at 72 hours of admission were also calculated by the Mann-Whitney test, which was higher in non-survivors (mean SOFA = 12.34, mean APACHE II = 25.74) as compared to survivors (mean SOFA = 10.89, mean APACHE II = 22.09), which was statically significant (p-value < 0.0001).

The cardiac biomarker NT-pro-BNP (mean ± SD) calculated by the Mann-Whitney test was found to be higher in non-survivors (503.24 ± 269.3) as compared to survivors (503.24 ± 269.3) (p-value < 0.0001), indicating an increase in cardiac enzyme showing the myocardium involvement in sepsis and septic shock [8].

FBS, PPBS, and HbA1C were analyzed by the unpaired Student's T-test. The mean ± SD of FBS in the survivors was 164.2 ± 68.9 mg/dl. In the non-survivors, the FBS was 173.5 ± 70.1 mg/dl; however, no statistically significant association was found (p = 0.466). The mean ± SD of PPBS in the survivors was 279.2 ± 66.9 mg/dl. In the non-survivor group, the PPBS was 293.5 ± 69.1 mg/dl, and there was no statistically significant association (p = 0.254). The mean ± SD of HbA1C level in survivors was 8.3 ± 1.2%, and in the non-survivors group, the HbA1C level was 8.5 ± 1.3%, which was statically not significant (P = 0.388) (Table 1).

## Discussion

Patients with T2DM having impaired blood glucose levels have the best environment for microorganism growth that could lead to a life-threatening condition called sepsis leading to septic shock. Frequently, these patients suffer from diastolic dysfunction, and diastolic dysfunction is an important early mortality indicator in such patients; it predicts the early mortality of the admitted patients, even after adjusting the other mortality indicators like SOFA score, APACHE II score, low urine output, and low oxygen saturation. Other independent mortality indicators do not show any correlation with FBS, PPBS, and HbA1C [6].

This study shows that patients with diabetes mellitus as a comorbidity have the highest association with diastolic dysfunction relative to other comorbidities. Hypertension has the second highest association, chronic kidney disease has the third, and chronic liver disease has the fourth highest association. However, our study included diabetes mellitus with other comorbidities like hypertension, chronic kidney disease, and chronic liver disease, hence the association of sepsis and diabetes cannot be fully explained. It is doubtful that other systemic complications might have enhanced the left ventricular dysfunction. However, in our study, we have excluded cases of coronary artery disease who have regional wall motion abnormalities, hence ischemic heart disease cannot be commented with the prevalence of diastolic dysfunction.

In our study, 132 patients were enrolled, though our sample size calculated was 109. We included 132 cases, as the expected total number of patients over the 18 months was 150, after the inclusion criteria and exclusion criteria.

All of the patients were treated according to surviving sepsis guidelines [4]. All these patients underwent transthoracic echocardiography and the diastolic dysfunction parameters were assessed and the left ventricular ejection fraction was calculated [5]. Our study results showed that the higher the E/A ratio, the higher the grade of LVDD and the early mortality rate with an increase in the SOFA score at 72 hours, indicating higher end-organ impairment compared to the lower E/A ratio.

After incorporating all independent clinical and echocardiographic variables, the most robust independent predictor was found to be a reduced septal e' wave. Following this, in descending order of significance, the variables that predicted survival included the SOFA score, APACHE II score, lowest pH, and lowest hemoglobin (g%) (p-value < 0.01).

Since the ground-breaking research by Parker and colleagues who employed cineangiography over two decades ago to reveal the prevalence of systolic dysfunction in septic shock, the intriguing connection between this dysfunction and improved survival rates has remained shrouded in mystery [10]. Subsequent studies utilizing echocardiography have also confirmed a significant occurrence, ranging from 20% to 60% of reduced ejection fraction (EF) in septic shock [11]. Importantly, this reduction in EF appears to be reversible in patients who successfully recover. However, in this study, we included all patients who had sepsis and septic shock, and the left ventricular ejection fraction (EF %) was calculated as a mean ± SD value of 55 ± 8 in survivors and 57 ± 12 in non-survivors, which was not significant. Almost all the patients had isolated diastolic dysfunction.

The cardiac biomarker NT-pro-BNP was the highest in non-survivors, with a mean ± SD value of 503 ± 269.3 pg/ml, with lower NT-pro-BNP in survivors with mean ± SD of 286.40 ± 206.9 pg/ml, calculated by Mann-Whitney test, which shows left ventricular heart failure is more prevalent in death group, i.e., those who have diastolic dysfunction with sepsis [12]. However, the cardiac biomarker Trop-I did not show any significant increase, suggesting even at very myocardium stress conditions, myocardium wall necrosis or apoptosis is minimal, showing that coronary artery perfusion and myocardium perfusion are maintained in severe sepsis [13]. Our study also found that the 28-day mortality with diastolic dysfunction in sepsis and septic shock in diabetes mellitus patients have significant results (p < 0.001), indicating that with a higher E/A ratio or higher grade of diastolic dysfunction, the ICU mortality is highest and have the shortest duration of ICU stay. In patients with grade 3 diastolic dysfunction, the mortality was 100%, with a mean ± SD of the duration of ICU stay of 6.2 ± 2.48, compared to other grades. Parker et al. have studied by cineangiography that systolic dysfunction is common in septic shock and paradoxically has better outcomes, but the study has shown sepsis-induced left ventricular dysfunction [14,15]. In our study with a total of 132 patients, there were 13 (100%) mortalities in grade 3 LVDD, 19 (86.37%) in grade 2, 41 (73.22%) in grade 1, and 15 (36.7%) mortalities in normal LVDD, showing the lowest mean of ICU stay, respectively, calculated by the chi-square test, which showed a significant difference, suggesting that there is a rise in mortality at 28 days as the grades of diastolic dysfunction grade increases, indicating that diastolic dysfunction is a major important indicator for early mortality in sepsis.

The SOFA scores calculated at admission and at 72 hours of admission, comparing the live patients and dead patients, were statistically significant and were calculated by the Mann-Whitney test, showing the mean increase in SOFA score plays an important role in 28-day mortality and also with a higher grade of LVDD, the SOFA score increases, compared to other grades of LVDD. The APACHE II scores were calculated at the time of admission and at 72 hours of admission, and the results calculated by the Mann-Whitney test show significant results, showing a rise in the APACHE II score is an important predictor of mortality [16]. The cardiac biomarker CPK-MB also shows a significant result with mean ± SD of 5.143 ± 14.05, indicating an increase in cardiac enzyme shows the myocardium involvement in sepsis and septic shock [17].

## Conclusions

Diastolic dysfunction is the strongest independent early mortality predictor in severe sepsis and septic shock patients with diabetes mellitus. Many physicians are concerned about ventricular systolic function, cardiac output, and pressure, here our study shows that diastolic dysfunction plays an important role in contributing to early mortality, hence it should not be overshadowed. No correlation was detected between mortality and the mean levels of FBS, PPBS, and HbA1c. Further studies with large sample sizes are required to test whether the severity of grade of diastolic dysfunction is correlated with severe sepsis and studies for any therapeutic interventions that might improve and increase survival.
